# *Xist*/*Tsix* expression dynamics during mouse peri-implantation development revealed by whole-mount 3D RNA-FISH

**DOI:** 10.1038/s41598-019-38807-0

**Published:** 2019-03-06

**Authors:** Hirosuke Shiura, Kuniya Abe

**Affiliations:** 1 Technology & Development Team for Mammalian Genome Dynamics, RIKEN BioResource Research Center, 3-1-1 Koyadai, Tsukuba, Ibaraki 305-0074 Japan; 2 Department of Epigenetics, Medical Research Institute, Tokyo Medical and Dental University (TMDU), 1-5-45 Yushima, Bunkyo-ku, Tokyo 113-8510 Japan; 3 Faculty of Life and Environmental Sciences, University of Yamanashi, 4-4-37 Takeda, Kofu, Yamanashi 400-8510 Japan; 4 Graduate School of Life and Environmental Sciences, University of Tsukuba, Ibaraki, 305-8577 Japan

## Abstract

During peri-implantation development in mice, X chromosome inactivation (XCI) status changes dynamically. Here, we examined the expression of *Xist* and its antisense partner, *Tsix*, via whole-mount 3D RNA-FISH using strand-specific probes and evaluated XCI status. The results indicate that *Xist* expression disappears completely by embryonic day (E) 4.5 without *Tsix* activation in the ICM and that *Xist* re-expression occurs at E4.75 in some cells, suggesting that random XCI is already initiated in these cells. Intriguingly, epiblast cells exhibiting biallelic *Xist* expression were observed frequently (~15%) at E5.25 and E5.5. Immunostaining analysis of epigenetic modifications suggests that global change in epigenomic status occurs concomitantly with the transition from imprinted to random XCI. However, global upregulation of H3K27me3 modifications initiated earlier than other modifications, occurring specifically in ICM during progression of *Xist* erasure. Although both *Xist* expression and imprinted XCI are thought to be stable in the primitive endoderm/visceral endoderm and trophectoderm/extraembryonic ectoderm lineages, transient loss of *Xist* clouds was noted only in a subset of extraembryonic ectodermal cells, suggesting distinct features of *Xist* regulation among the three different embryonic tissue layers. These results will serve as a basis for future functional studies of XCI regulation *in vivo*.

## Introduction

In female mammals, one of the two X chromosomes is inactivated for gene dosage compensation between XX females and XY males^1^. This phenomenon, termed X chromosome inactivation (XCI), is regulated by several factors, such as the noncoding RNA *Xist* and its antisense sequence *Tsix*. *Xist* is exclusively expressed from the inactive X (Xi) and accumulates on it, leading to a chromosome-wide inactivation of gene expression^2–5^. *Tsix*, with its repressive effect on *Xist* expression, is expressed normally from the active X and is silenced on Xi^6,7^. Imprinted XCI occurs in preimplantation-stage embryos and *Xist* is essential for its initiation^8^. During this process, the paternal X (Xp) is preferentially selected as Xi according to a maternal imprint causing *Xist* repression on maternal allele^9–11^. The maternal imprint is now thought to be H3K27me3 modifications laid onto maternal X during oogenesis^12^. The imprinted XCI is then erased in the embryonic lineage, and XCI is resumed later as random XCI, in which Xi is chosen randomly. The erasure of imprinted XCI initiates in the inner cell mass (ICM) of early blastocysts. This is accompanied by the loss of *Xist* RNA accumulation, EED/EZH2 association and histone H3 lysine 27 trimethylation (H3K27me3) modifications from the Xp, and derepression of genes that are subjected to inactivation on the Xp^13–16^. During this erasure process, epigenetic memories for imprinted XCI are thought to be erased and both X chromosomes become epigenetically equivalent. Random XCI takes place after this imprinted XCI erasure. Although these event sequences have been described^17^, the precise timing of XCI erasure and initiation of random XCI during the development of peri-implantation embryos *in vivo* is not understood fully.

The reasons for studying the precise kinetics of XCI during embryonic development are at least twofold. First, basic information on the dynamics of XCI will provide clues that will contribute to understanding the regulatory mechanisms that operate *in vivo*. Traditionally, the *in vitro* differentiation system of embryonic stem (ES) cells has been used extensively in studying XCI. Despite its great experimental advantages, the *in vitro* ES cell system cannot cover all aspects of the XCI dynamics that occur *in vivo*. For example, imprinted XCI does not exist in the ES cell system. To reevaluate the usefulness of the ES cell system and to understand the XCI phenomenon in general, studies of embryos developing *in vivo* are essential, as they add complementary knowledge to the data accumulated from *in vitro* studies. Second, changes in XCI status are likely to be coupled with epigenomic or nuclear reorganization in developing peri-implantation mouse embryos. ICM and mouse ES cells (mESCs) represent a ground state (naïve state) of pluripotency, whereas epiblasts of postimplantation-stage embryos or epiblast stem cells (EpiSCs) correspond to a “primed” pluripotent state^18^. XCI is one of the key features of EpiSCs. In contrast with female mESCs, where the two X chromosomes are both active, a random XCI operates in female EpiSCs. It is becoming increasingly clear that there are significant differences in epigenetic status or an “epigenetic barrier” between the naïve and primed states of pluripotent stem cells^19–21^, and that the imprinted XCI–random XCI conversion that takes place in peri-implantation mammalian embryos might be a reflection of epigenomic reorganizations that are not restricted to X chromosomes. Therefore, we believe that the XCI status could be a useful indicator of large-scale epigenomic reprogramming events that have remained unexplored to date.

*Xist* RNA clouds or coatings (i.e., the accumulation of *Xist* RNA over the entire Xi) are one of the indicators of whether cells are in XCI state^22–24^, and the accumulation of *Xist* RNA is lost during imprinted XCI erasure^13,14,16^. As *Xist* RNA is essential for the establishment of XCI, re-expression of *Xist* is thought to be the sign of random XCI commencement. However, the repression of *Xist* itself might not represent an active state of the X chromosome, because it is known that the expression/repression status of *Xist* does not necessarily coincide with the expression status of other X-linked genes. For example, it has been reported that *Xist* expression is dispensable for X inactivation in mouse embryonic fibroblasts (MEFs)^25^ or in developing primordial germ cells (PGCs)^26^. Moreover, it has been demonstrated that several X-linked genes located on the imprinted Xi are reactivated even in the presence of *Xist* coatings^15,16^. Therefore, it is necessary to examine both *Xist* repression and activation of X-linked gene(s) to judge whether XCI reversal occurs or not. Among the numerous X-linked genes, we have paid particular attention to *Tsix*, an antisense RNA partner of *Xist* that plays a repressive role in *Xist* expression^7,27,28^. It is known that continued expression of an inducible *Tsix* allele causes stable repression of *Xist* in imprinted XCI during preimplantation development^28^. However, whether *Tsix* has an active role in induction of *Xist* repression during imprinted XCI erasure *in vivo* is not fully understood. Furthermore, in female ES cells, *Tsix* is expressed at high levels from both alleles, and it is proposed that this biallelic *Tsix* expression induces changes in histone modifications along the *Xist* locus that ensure the epigenetic equivalency of both X chromosomes in undifferentiated pluripotent cells^29^, implying that the cells that have undergone X reactivation will show biallelic expression of *Tsix* both *in vitro* and *in vivo*. Therefore, we decided to track the kinetics of both *Xist* and *Tsix* expressions to know whether *Tsix* reactivation trigger the initiation of *Xist* repression and make a careful estimate of XCI status during XCI reversal and random XCI establishment. In addition, as *Tsix* reactivation would not necessarily reflect the overall state of the X chromosome, expression statuses of other X-linked genes (*Lamp2* and *Pgk1*) were also examined to confirm progression of X reactivation at the developmental stage when biallelic expression of *Tsix* became predominant. To perform a quantitative expression analysis in peri-implantation-stage embryos, we used whole-mount RNA fluorescence *in situ* hybridization (FISH), which had been successfully utilized for study of X reactivation in PGCs^26^. RNA-FISH is better suited to assessing the on-and-off state of transcription because it can detect nascent RNA and not the steady-state level of RNA. Furthermore, the whole-mount technique, which does not destroy embryonic structures, enables the unambiguous detection of RNA expression in different embryonic lineages, such as the trophectoderm/extraembryonic ectoderm (TE/ExE), primitive endoderm/visceral endoderm (PE/VE), and ICM/epiblast lineages.

The results presented here demonstrate *Xist*/*Tsix* dynamics during peri-implantation development at an unprecedented resolution, implying the period of imprinted XCI erasure and the timing of random XCI commencement in the ICM/epiblast lineage *in vivo*. Moreover, we found differences in *Xist*/*Tsix* dynamics and changes in epigenetic status among the three different lineages of embryos. The information regarding *Xist*/*Tsix* dynamics *in vivo* obtained in this study will serve as a basis for future functional studies on XCI regulation.

## Results

### Kinetic changes in *Xist*/*Tsix* status during development of the embryonic lineage

**Figure 1:**
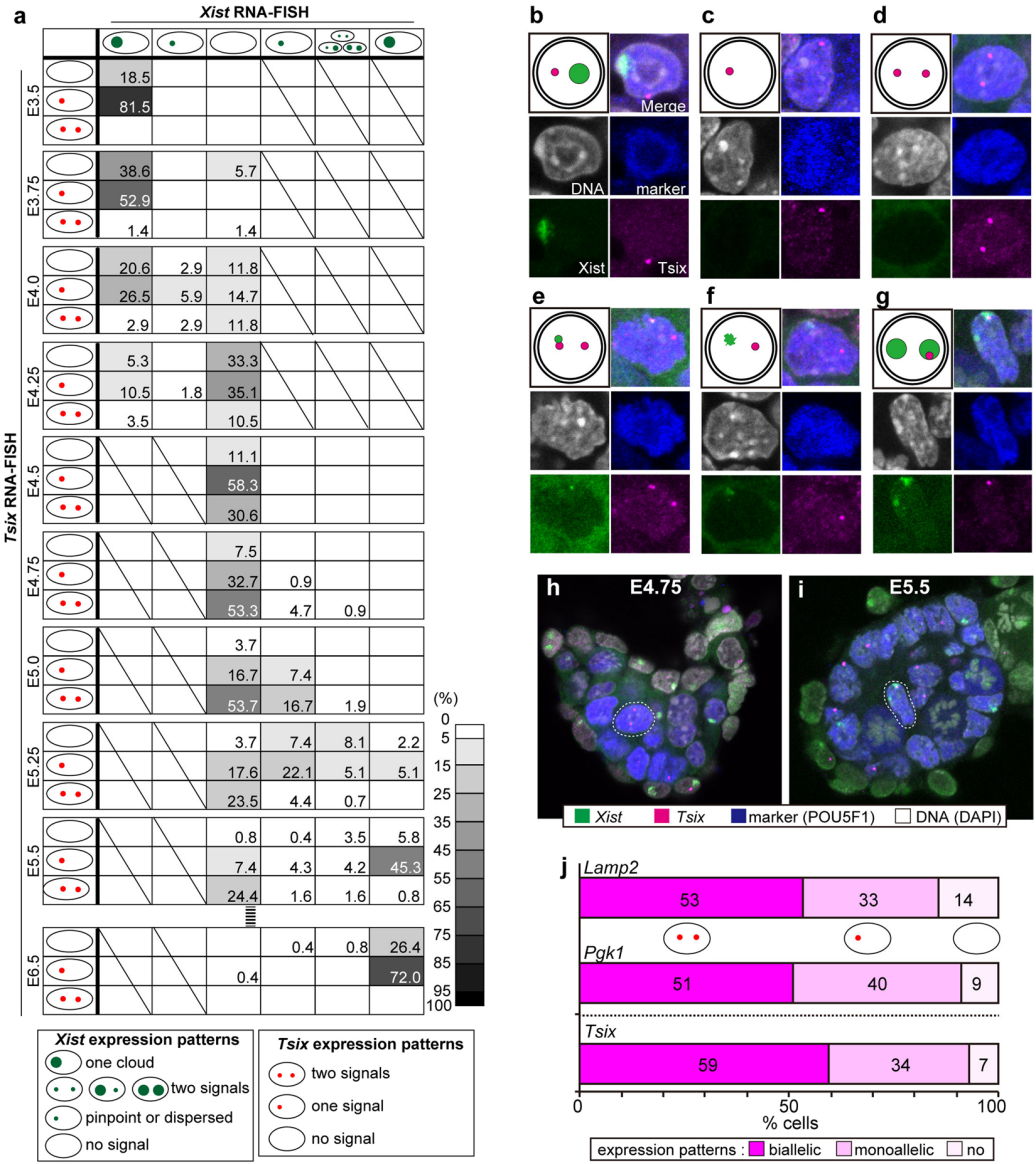
*Xist*/*Tsix* expression kinetics in the embryonic cell lineage. (**a**) The matrices represent the *Xist* and *Tsix* expression patterns of embryonic-lineage cells at each developmental stage. The rows represent *Tsix* expression patterns (no signal, one, or two signals) and the columns represent *Xist* expression patterns (one cloud, single pinpoint or dispersed signal, no signal, or two signals). The numbers displayed at the intersections of rows and columns indicate the percentage of cells with the *Tsix* and *Xist* expression pattern indicated by the row and the column, respectively. (**b**–**g**) Representative images of cells showing different patterns of *Xist* (green)/*Tsix* (magenta) expression. The blue and white colors indicate lineage-marker expression and nuclear DNA staining, respectively. (**b**) One *Xist* cloud and a single *Tsix* signal; (**c**) no *Xist* and a single *Tsix* signal; (**d**) no *Xist* and two *Tsix* signals; (**e**) one *Xist* pinpoint signal and two *Tsix* signals; (**f**) one *Xist* dispersed signal and a single *Tsix* spot; and (**g**) two *Xist* clouds and a single *Tsix* signal. (**h**,**i**) Examples of single optical sections of E4.75 (**h**) and E5.5 (**i**) embryos. The cells enclosed by a dotted circle correspond to the cells shown in (**d**,**g**), respectively. (**j**) The expression patterns of two ordinary X-linked genes, *Lamp2* (77 nuclei in 3 embryos) and *Pgk1* (57 nuclei in 2 embryos), and *Tsix* in the embryonic cell lineage at E4.75.

**Figure 2:**
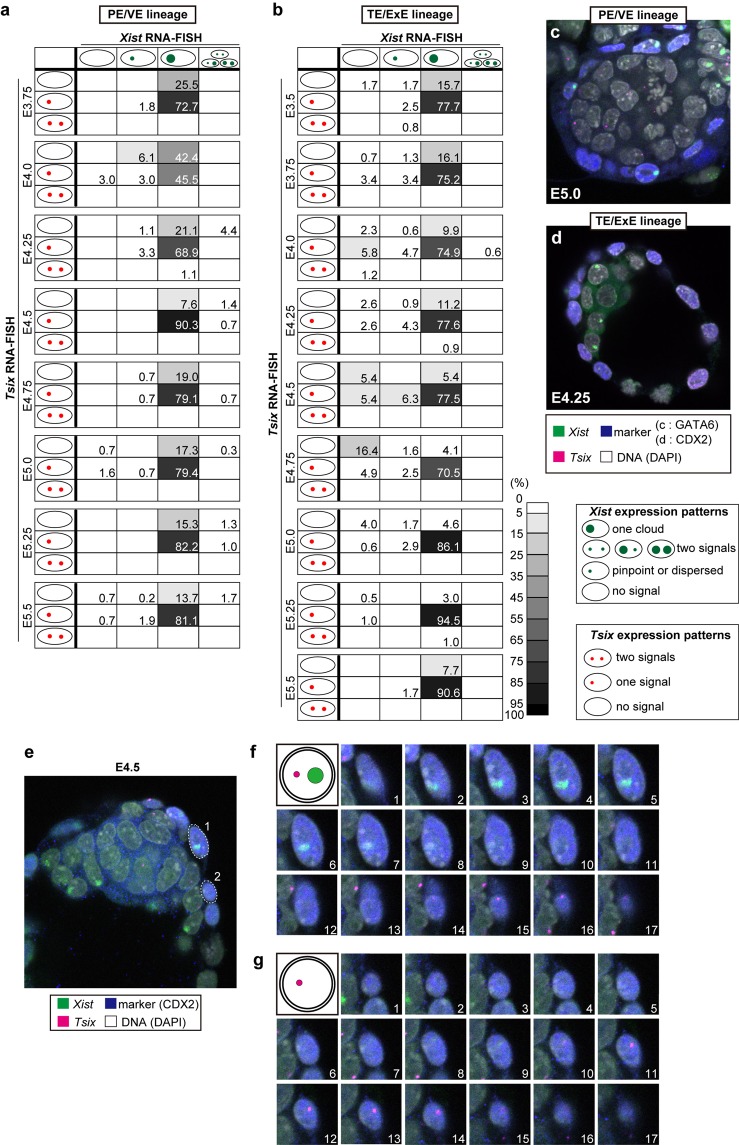
*Xist*/*Tsix* expression kinetics in extraembryonic lineages. (**a**,**b**) The matrices represent *Xist* and *Tsix* expression patterns in PE/VE lineage cells (**a**) and TE/ExE lineage cells (**b**) in each developmental stage, respectively. (**c**,**d**) Representative images of *Xist* (green)/*Tsix* (magenta) RNA-FISH combined with GATA6 (for PE/VE lineage (**c**)) and CDX2 (for TE/ExE lineage (**d**)) immunofluorescence with nuclear DNA staining (white). (**e**) Image of an E4.5 embryo. Cell 1 exhibited a *Xist* cloud, whereas cell 2 was negative for the *Xist* signal. (**f**,**g**) Serial sections of cells 1 (**f**) and 2 (**g**) respectively.

*Xist* and *Tsix* expression in ICM/epiblast, PE/VE and TE/ExE lineages were examined by using strand-specific RNA-FISH (see Methods for details of the strand-specific probe preparation) combined with immunofluorescence against those cell lineage markers during a period between E3.5 and E5.5 at intervals of 6 h (Figs 1, 2, Table 1 and Supplementary Fig. S1a). Embryonic lineage cells were marked by an antibody against the pluripotent cell marker POU5F1 (OCT3/4) for E3.5 blastocysts and for embryos sampled between E4.75 and E5.5. As POU5F1 expression was observed in both the primitive endoderm and the epiblast at stages from E3.75 to E4.5, anti-NANOG was used instead to stain embryonic lineage cells (Supplementary Fig. S1a). Images were acquired on a confocal microscope, and z-stack images were used to analyze *Xist*/*Tsix* expression patterns in each cell of the whole embryo (Fig. 1b–i; see Supplementary Video S1). We used this three-dimensional (3D) whole-mount RNA-FISH technique^26^ to determine the kinetic changes in *Xist*/*Tsix* expression in almost all the cells that constituted entire embryos at E3.5–5.5, and the analysis was also performed at E6.5 (Table 1, 4,127 nuclei in 107 embryos in total). *Xist*-positive cells were classified into four categories: cells with one *Xist* cloud per nucleus, cells with single pinpoint or dispersed signals, cells with two *Xist* signals, or cells with no *Xist* signal. *Tsix*-positive cells were categorized into cells with two, one, or no signals. To understand the relationships between the expression patterns of *Xist* and *Tsix* in the same individual cells, a matrix illustrating proportional data (%) for each category of *Xist*- or *Tsix*-positive cells was constructed (Fig. 1a).

**Table 1:** Numbers of cells (embryos) examined in three different cell lineages at each stage.

Stage	Embryonic lineage, cells (embryos)	PE/VE lineage, cells (embryos)	TE/ExE lineage, cells (embryos)
E3.50	27 (3)	—	121 (4)
E3.75	70 (4)	55 (4)	149 (4)
E4.00	34 (3)	66 (5)	171 (5)
E4.25	57 (5)	90 (4)	116 (4)
E4.50	36 (3)	144 (4)	111 (4)
E4.75	107 (5)	153 (3)	169 (4)
E5.00	108 (4)	306 (5)	173 (4)
E5.25	136 (3)	314 (4)	199 (5)
E5.50	258 (4)	417 (4)	286 (4)
E6.50	254 (2)	—	—
	1087 (36)	1545 (33)	1495 (38)

Total: 4127 cells (107 embryos).

In E3.5 blastocysts, more than 80% of ICM cells possessed one *Xist* cloud and one pinpoint *Tsix* signal per nucleus (Fig. 1b), indicating that most ICM cells were still subjected to imprinted XCI at this stage. From E3.75 onward, *Xist* cloud signals began to disappear and cells showing biallelic expression of *Tsix* began to appear (Fig. 1c,d).

At E4.5, all epiblast cells were *Xist* negative. A large proportion of the cells (58%) showed a single *Tsix* signal, whereas 11% and 31% of cells exhibited no or two *Tsix* signals, respectively. This indicates that the erasure of *Xist* precedes the derepression of *Tsix* from the silent allele and that *Tsix* activation might not be a prerequisite for the disappearance of *Xist*. The numbers of cells showing *Tsix* biallelic expression increased after E4.5, whereas the numbers of *Tsix* single-positive cells decreased. Most cells (>53%) exhibited biallelic expression of *Tsix* and no expression of *Xist* (Fig. 1h) at E4.75 and E5.0, and the numbers of cells showing *Tsix* biallelism then decreased from 72% at E5.0 to 29% at E5.25. These results suggest that reactivation of the silent *Tsix* allele occurred increasingly after E4.5 and plateaued at E4.75–5.0. At E4.75, the proportions of the cells showing biallelic expression of *Lamp2* and *Pgk1* were almost the same as that of the cells exhibiting *Tsix* biallelic expression (Figs 1j and S2). The result indicates that reactivation of “ordinary” X-linked genes, in addition to *Tsix*, was also progressing at this stage. A small number of cells in the epiblast had already begun to show weak *Xist* signals at E4.75 and the proportion of such cells increased thereafter, indicating the random XCI process had already begun in those cells. These *Xist* weakly positive cells were not clustered or positioned in any specific region of the epiblast. These results suggest that switching from the XCI reversal to random XCI had already been initiated in some nuclei at E4.75–5.0 before all the epiblast cells had completed XCI reversal. The numbers of *Xist*-positive cells increased further so that at E5.0, 26% of the epiblast cells showed weak *Xist* expression as a pinpoint signal (Fig. 1e), whereas more than half of the cells (55%) exhibited *Xist* signals at E5.25. Although most of the cells showed dispersed *Xist* signals (Fig. 1f), cells displaying *Xist* clouds began to appear from this stage onward, suggesting that the expression level of *Xist* increased gradually between E4.75 and E5.25. During this period, the proportion of cells with two *Tsix* signals and one *Xist* signal decreased, whereas the numbers of cells with one *Tsix* and one *Xist* signal increased, implying that elevated expression of *Xist* seems to cause *Tsix* silencing. At E5.5, a large proportion (>50%) of the cells showed one *Xist* cloud. *Xist* /*Tsi*x expression patterns in embryonic lineage at E6.5 were also examined and the result showed that almost all of the cells displayed a single *Xist* cloud with or without one *Tsix* signal and cells with biallelic *Tsix* expression were absent (Fig. 1a and Table 1). This suggests that Xi had already been chosen in almost all the epiblast cells by E6.5.

Intriguingly, a significant number of epiblast cells exhibited biallelic expression of *Xist* at E5.25 (13.9%) and E5.5 (9.3%) (Fig. 1a,g,i). Cells with two *Xist* clouds were also detected, and the frequency of such cells increased at E5.5 (Figs 1g,i and S3). Combined analysis of RNA and DNA-FISH can directly address whether those cells were normal diploid or tri-/higher-ploid cells, but such combined analysis is not compatible with our whole-mount protocol (see Discussion). Instead, we reasoned that potential triploid cells exhibit two *Xist* clouds and one *Tsix* signal on the three different positions and looked for such cases. In this analysis, we hardly observed the cells in which one or more *Tsix* signals were localized distantly from two *Xist* clouds. In fact, there was only one cell showing potential triploidy or higher ploidy in the epiblast cells at E5.25 (1 cell in 136 cells observed), as well as one such cell at E5.5 (1 in 258 cells). In both cases, the cell exhibited two *Tsix* signals and one *Xist* signal localized distantly from *Tsix* signals. Therefore, although the possibility of aneuploidy cannot be ruled out completely, we believe that it is highly likely that those cells exhibiting the two *Xist* signals observed in this study were normal diploid cells.

### *Xist* expression appears to be stable in the PE/VE lineage during peri-implantation development

Most of the cells (88–98%) in the PE/VE lineage marked by GATA6 expression (Supplementary Fig. S1a) exhibited one *Xist* cloud per nucleus throughout the developmental period examined here (Fig. 2a,c). This result suggests that the imprinted repression of maternal *Xist* and paternal *Xist* expression are basically maintained in this lineage from E3.5 to E5.5. However, pinpoint signals for *Tsix* were lost specifically at E4.0 in nearly 50% of the cells. Loss of *Tsix* expression of this kind has never been observed in ExE cells. At E4.0 and E4.25, some cells (12.1% at E4.0 and 4.4% at E4.25) exhibited weak or no signals for *Xist*. As differentiation of the PE and epiblasts could not be delineated clearly by the lineage markers NANOG and GATA6 at this stage (Supplementary Fig. S1b), PE/VE cells with weaker *Xist* expression might represent precursor cells of the epiblast lineage, in which *Xist* will disappear.

### Transient loss of *Xist* clouds in a subset of ExE cells around the implantation stage

The TE or ExE cells were marked by CDX2 expression (Supplementary Fig. S1a and Supplementary Video S2). At all stages examined, most of the TE/ExE cells (70–95%) possessed one *Xist* cloud and a single *Tsix* spot (Fig. 2b,d–f), suggesting that the imprinted XCI persisted throughout the development of this lineage. However, careful examination of the expression of *Xist* revealed that cells with no *Xist* signals were present and that the number of these cells increased transiently, reaching a peak at around E4.75 (21% of all the ExE cells showed no *Xist* expression; Fig. 2b,e,g). These *Xist*-negative cells were not clustered or positioned in a specific region of ExE tissues. Most of these *Xist*-negative cells had either a single or no *Tsix* signal, and very few cells showed two *Tsix* signals. This suggests that the transient loss of *Xist* expression occurs independently of the status of *Tsix* expression from the Xi.

### Epigenetic dynamics during peri-implantation development

**Figure 3:**
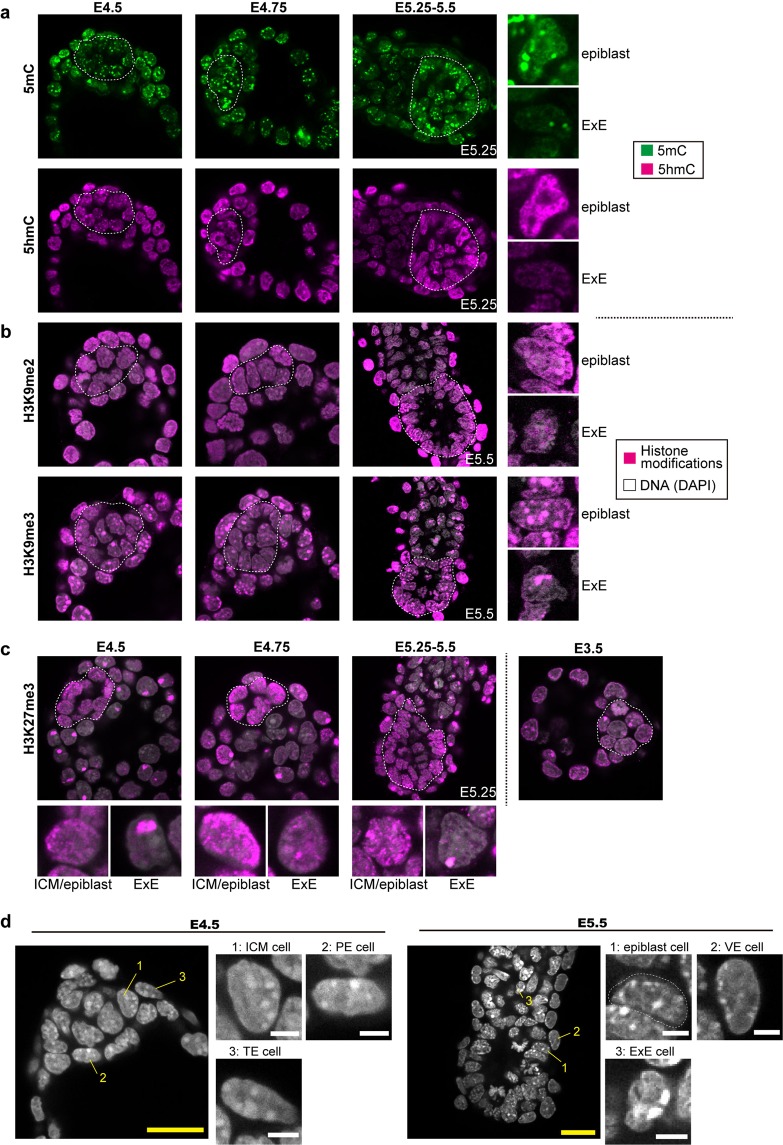
Epigenetic dynamics during peri-implantation development. (**a**–**c**) Immunofluorescence analysis of epigenetic modifications in peri-implantation embryos using antibodies against 5 mC, 5 hmC (**a**) H3K9me2, H3K9me3 (**b**) and H3K27me3. (**c**) The green and magenta colors indicate immunofluorescence signals of 5mC and each other modification, respectively and white dotted circles indicate the location of the ICM/epiblast lineage cells. 5 mC and 5 hmC staining were performed in the same embryos. The images of H3K9me2, H3K9me3, and H3K27me3 are overlapped with nuclear staining (white). Magnified views of epiblast and ExE cells at E5.25–5.5 (**a**,**b**) and E4.5, 4.75, and E5.25–5.5 (**c**) are shown on the right side (**a**,**b**) and bottom of each image (**c**) respectively. (**d**) The images of DAPI staining at E4.5 and 5.5. Magnified views of 1: ICM/epiblast, 2: PE/VE and 3: TE/ExE cells at each developmental stage are shown. The yellow and white scale bars indicate 25 and 5 μm, respectively.

To examine global epigenomic changes during peri-implantation development, we performed immunofluorescence analysis on modifications of DNA, i.e., 5-methylcytosine (5 mC) and 5-hydroxymethylcytosine (5 hmC) (Fig. 3a) as well as histone modifications such as di- and trimethylation of histone H3 lysine9 (H3k9me2 and me3) (Fig. 3b) and trimethylation of histone H3 lysine 27 (H3K27me3) (Fig. 3c).

As shown in Fig. 3a,b, the levels of 5 mC, 5 hmC, H3K9me2 and H3K9me3 in ICM/epiblasts were comparable with or somewhat lower than those in ExE at E4.5 and E4.75, whereas the signals appeared to be more prominent in epiblasts relative to ExE at E5.25–5.5. The results suggest that changes in the four epigenetic modifications showed a similar trend. Namely, global levels of the modifications increase after E4.75.

In contrast to the four modifications mentioned above, the H3K27me3 modification behaves differently (Fig. 3c). At E3.5, H3K27me3 staining signals were detected as a single, heavy deposit probably corresponding to Xi over a relatively weak background in each ICM and TE cell. However, at E4.5, H3K27me3 modifications were globally upregulated specifically in ICM and this global upregulation was continuously observed at E4.75 and E5.25–5.5. Therefore, upregulation of global H3K27me3 level in epiblast initiated earlier compared to the increase in other four modifications. Interestingly, the H3K27me3 staining was seen as particulate structures distinct from chromocenters as shown in Fig. 3c.

In addition, we noted that the heterochromatin organization, revealed by DAPI staining, changed dramatically in different cell lineages at different developmental stages (Fig. 3d). The number and morphologies of DAPI-dense chromocenters were roughly similar to each other between ICM, PE, and TE cell lineages at E4.5. However, at E5.5, each cell in different lineages appeared to develop unique heterochromatin organization. In epiblasts, chromocenters were distributed over the entire nucleus, whereas VE lineage cells were characterized by smaller chromocenters distributed along the nuclear membrane and ExE lineage cells showed relatively few and large chromocenters. These observations suggest that distinct epigenetic status and chromatin organizations characteristic to these three lineages are established in a relatively short period during peri-implantation development.

## Discussion

**Figure 4:**
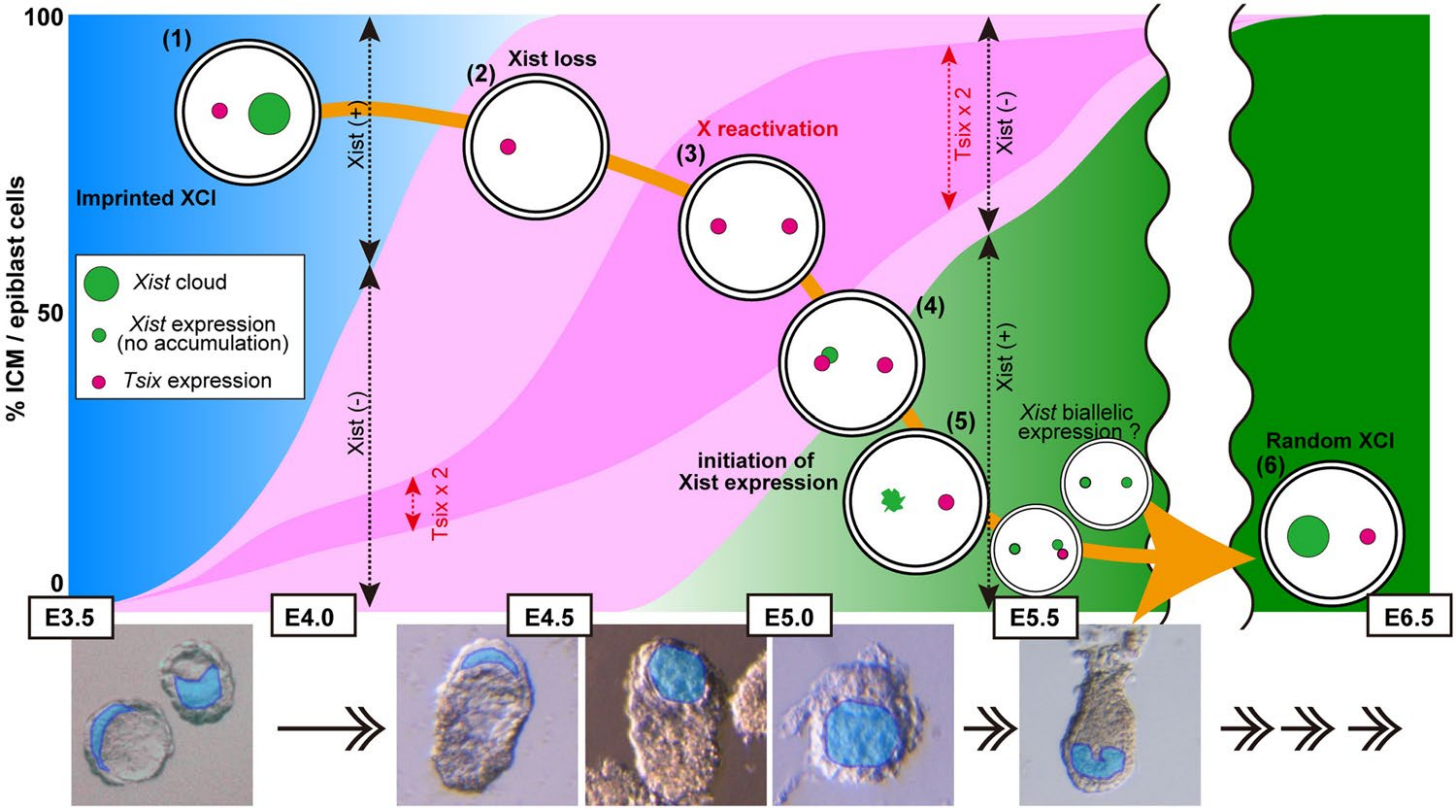
A model of *Xist*/*Tsix* expression dynamics during the development of peri-implantation mouse embryos. (1) Imprinted XCI; the Xp is inactivated and a *Xist* cloud is observed on it, whereas *Tsix* shows a single spot on the maternal X (blue region). (2) Cessation of *Xist* expression commences at E3.75, and the *Xist* cloud disappears by E4.5 (light pink region). (3) The silenced *Tsix* gene is derepressed on the Xp in the embryonic lineage. Most cells show biallelic *Tsix* expression between E4.75 and E5.0 (deep pink region). Cells undergoing X-reactivation are included in this region. (4) A single *Xist* spot, rather than a cloud, appears in a small percentage of cells showing *Tsix* biallelic expression (green region). (5) A single *Xist* spot or *Xist* cloud is observed in cells showing monoallelic *Tsix* expression. Generally, *Xist* and *Tsix* are expressed in a mutually exclusive manner on X chromosomes. However, a significant number of cells exhibit double *Xist* signals. These ‘double *Xist*’ cells appear transiently in E5.25–5.5. (6) Random XCI is fully established by E6.5.

We traced *Xist*/*Tsix* expression during peri-implantation embryonic development and attempt to address assessment of XCI dynamics *in vivo* using whole-mount 3D RNA-FISH (Fig. 4). The results showed that *Xist* erasure began at E3.75 and was completed by E4.5, whereas the cells that exhibited biallelic expression of *Tsix* and no expression of *Xist* became dominant at the following stage between E4.75 and E5.0. The expression patterns of two X-linked genes, *Pgk1* and *Lamp2*, were also examined at E4.75. Proportions of cells showing biallelic expression of these genes were very similar to that of cells exhibiting *Tsix* biallelic expression, indicating that X reactivation certainly occurred for X-linked genes other than *Tsix*. Borensztein *et al*. clearly demonstrated that the timing of reactivation varied among individual X-linked genes and classified X-linked genes into ‘early reactivated (reactivated from E3.5)’, ‘late reactivated (from E4.0)’ and ‘very late reactivated’. The ‘very late reactivated’ genes were not reactivated even at E4.0^16^. *Pgk1* gene is classified as late reactivated gene, and not fully reactivated at E4.0. Here, we demonstrated that *Pgk1*, one of the late reactivated genes, was activated in about half of the embryonic lineage cells at E4.75. *Tsix* was activated in only ~15% of the ICM cells at E4.0–E4.25 and ~50% at E4.75, suggesting that *Tsix* is categorized into the ‘late reactivated’ genes or possibly later than “late reactivated” genes. Combined, reactivation of *Pgk1*, *Tsix* and *Lamp2* likely occurs at the late-to-very late stage of XCI reversal. The cells exhibiting two *Tsix* without *Xist* signals may not always mean X-reactivated state. Nevertheless, as all of X-linked genes including *Tsix* should be biallelically expressed in the cells subjected to XCI reversal, biallelic expression of *Tsix* without *Xist* is, at least, necessary condition for X-reactivated state. Therefore, based on the *Xist*/*Tsix* expression pattern as well as results of two other X-linked genes, we think that the proportion of X-reactivated cells in the embryonic lineage appears to peak at E4.75–5.0. We found that, already at E4.75, a few epiblast cells exhibited weak *Xist* expression while the same cells continued to show *Tsix* biallelism. Such cells are considered to have initiated random XCI. These cells showing ‘early’ *Xist* expression were not clustered or positioned spatially in specific regions of the embryos. The numbers of these cells further increased until E5.0, and then *Tsix* biallelic expression declined at later stages.

At E6.5, almost all of the cells displayed a single *Xist* cloud and cells with biallelic *Tsix* expression were absent. Using the same RNA-FISH technique, it was demonstrated that there was no epiblast cell exhibiting biallelic expression of a X-linked gene, *Mecp2*, at E6.5^30^. These results suggest that random XCI is established by E6.5. Previous cytogenetic studies based on Kanda staining^31^ or DNA replication timing^32^ demonstrated that random XCI is established by E5.5 or around E6.5, respectively. Those results were generally consistent with ours based on *Xist*/*Tsix* expression. The slight differences in the timing found between studies might have been caused by differences in the genetic background of the strains used or could be a reflection of the use of different indices representing different stages of the random XCI process.

We also found that X-reactivation seemed to progress in a cell division-independent manner. Although NANOG-positive ICM cells did not proliferate actively from E3.75 to E4.5 (Table 1), imprinted XCI reversal can progress at these stages. Conversely, after E4.75, when the epiblast cells resume *Xist* expression to establish random XCI, cell proliferation was accelerated dramatically (Table 1). Such a XCI reversal event is also observed during PGC development and is accompanied by genome-wide epigenetic reprogramming^26,33,34^. Here, we demonstrated that a large-scale change in epigenetic modifications and in chromatin organizations also occurred between E3.5 and E5.5, in particular during E4.75 and E5.25–5.5 when *Xist* accumulation on one of the two X chromosomes is progressively established. Interestingly, H3K27me3 upregulation in the ICM/epiblast lineage, in contrast to all the other epigenetic modifications examined, was observed between E3.5 and E4.5. This developmental period exactly matches the period of *Xist* erasure. It has been reported that H3K27me3 is required for *Tsix*-independent *Xist* repression in naïve ES cells^35^. In addition, Inoue *et al*. demonstrated that H3K27me3 is required for maternal *Xist* silencing in imprinted XCI during pre-implantation development^12^. Therefore, it is possible that paternal *Xist* repression observed in ICM/epiblasts may be caused by H3K27me3 modifications laid onto paternal *Xist* regulatory regions after E3.5. These results indicate that the major de- and reprogramming events of the epigenomic status occur at around the peri-implantation stage, and thus the conversion of XCI regulation from imprinted to the random process seems to be one of those major epigenomic events.

Here we showed that *Xist* erasure occurred without any activation of *Tsix* from the Xi, suggesting that *Tsix* expression is not involved in initiating the erasure of the *Xist* RNA. Forced induction of *Tsix* expression caused *Xist* repression in *cis*, resulting in Xi reactivation in a lineage- and stage-specific manner^28^. In contrast with this ‘gain-of-function’ type of experiment, Maclary *et al*. showed that XCI reversal in the ICM could occur in the absence of functional *Tsix*, suggesting that *Tsix* is dispensable for X chromosome reactivation at this stage^36^. Moreover, Payer *et al*. observed a loss of H3K27me3 enrichment on the Xi of epiblast cells in *Tsix*-null mutant embryos, although the loss was significantly delayed in the mutants compared with normal embryos^37^. The results of these two studies based on *Tsix* null mutants were consistent with our findings. Thus, the onset of the loss of inactive markers of the Xi can occur in the absence of *Tsix* expression. Pasque *et al*. demonstrated that *Tsix* expression is dispensable for XCI reversal during the reprogramming of MEFs to induced pluripotent stem cells (iPSCs)^38^. Therefore, is *Tsix* not essential for X chromosome reactivation at all? Considering that the X reactivation process can be divided into at least two phases—the initiation of release from the inactive state and the establishment of epigenetic equivalency between the two X chromosomes—*Tsix* is probably not involved in the former phase, but is likely to be involved in the latter, thus assuring the epigenetic parity of both X chromosomes, as suggested by Navarro *et al*.^29^.

Intriguingly, here we showed that the biallelic expression of *Xist*, including two *Xist* clouds, could take place at the onset of random XCI during normal mouse embryonic development. In general, to examine the ploidy of those cells, a combined analysis of RNA- and DNA-FISH can be used. For the combined analysis, samples are fixed and anchored on a glass slide so that DNA-FISH images are superimposed to the recorded RNA-FISH signals. However, as the embryos examined in our whole-mount protocol were not anchored, we could not perform a combined analysis of RNA- and DNA-FISH. Alternatively, we searched potential triploidy or higher ploidy cells exhibiting one or more *Tsix* signals localized distantly from two *Xist* clouds. However, we rarely found such cases. Therefore, we think that the cells with two *Xist* clouds were likely to be normal diploid cells. This conclusion is also supported by *in vitro* studies performed by other groups: biallelic expression of *Xist* was detected in a subset of differentiating diploid ES cells^39–42^; live-cell imaging of the fluorescently tagged nascent Xi *in vitro* showed survival of some cells with two Xi signals^41^; and two *Xist* signals could be also detected in diploid ICM cells allowed to differentiate *in vitro*^40^. In addition, ectopic expression of *Xist* was also observed in epiblast cells of male embryos at the same stage (Supplementary Fig. S4) although frequency of detection was much lower in male (1.2 cells per embryo at E5.5 (n = 5)) than in female (6 cells per embryo at E5.5 (n = 4)). This trend is also true for differentiating ES cells^40,42^. Therefore, it is possible that such biallelic expression of *Xist* in female, and also ectopic *Xist* expression in male, can occur in the normal diploid cells during peri-implantation development in mice. Similar results have been reported recently by Sousa *et al*.^42^. At E4.75 when re-expression of *Xist* began to be observed in the ICM/epiblast cells of female embryos, the cells exhibiting biallelic expression of *Xist* already appeared. In addition, the percentage of the cells showing asymmetric expression of *Xist*, e.g. one pinpoint (or dispersed) and one cloud, was lower than that of the cells showing two *Xist* pinpoints (or dispersed) signals or two clouds (Supplementary Fig. S3). Therefore, it appears that the *Xist* biallelism is caused by simultaneous *Xist* activation from both alleles rather than an occasional up-regulation of the second *Xist* allele in the cells that have already initiated *Xist* expression from one allele. It was unexpected and intriguing observation, because *Xist* RNA accumulation on the future Xi is supposed to occur after determining the number of X chromosomes and election of the future Xi and Xa^43^. Since the present study only provides snap shots of initiation process of random XCI, longitudinal observations using different techniques will be needed to delineate how exactly random XCI initiates or to determine the fate of cells with *Xist* biallelic expression. Biallelic *Xist* activation can be found in a high proportion of cells during early development in human and rabbit which have no imprinted XCI mechanism^44^. Although these phenomena are superficially similar to the one in mice, *Xist* biallelism in mice may have different biological significance. Proportion of the epiblast cells exhibiting *Xist* biallelic expression in post-implantation epiblast of mice is low compared to those in human pre-implantation embryo (at most 15% in mice vs >80% in human) and cells showing *Xist* biallelic expression were only transiently detected (from E5.25 and E5.5), whereas *Xist* biallelism is observed in relatively long period of human preimplantation development, implying that the transient *Xist* biallelic expression may be harmful for mouse epiblast cells, thereby causing XCI on both X chromosomes. On the other hand, *Xist* accumulation does not induce chromosome-wide XCI in human preimplantation embryos^44^. Interestingly, as described above, ectopic expression of *Xist* in mice was observed at almost the same stage in both female and male epiblast cells. It is thus possible to speculate that such ectopic *Xist* expression in mice might be a reflection of large-scale changes in epigenomic states and/or in nuclear architecture that occur in both male and female embryos at a similar developmental stage corresponding to the beginning of random XCI establishment in female embryos.

Our *in vivo* analysis revealed that *Xist*/*Tsix* expression dynamics were significantly different among the three different embryonic lineages studied: ICM/epiblast, TE/ExE, and PE/VE. Interestingly, *Xist* expression was lost transiently in some ExE cells, from E4.0 to E5.0, reaching a peak of 21% at E4.75. One possible explanation for the loss of *Xist* clouds is that those cells were in early G1 phase because *Xist* clouds may not be observed in early G1 phase depending on pretreatment (permeabilization) of the cells prior to RNA-FISH^45^. As another explanation, we speculate that loss of *Xist* clouds is possibly linked to the change in regulatory mechanism of imprinted XCI in the ExE lineage. Imprinted Xp inactivation is thought to continue in ExE cells. However, the imprinted XCI can be reversed in a subset of female ExE cells that carry a paternally derived *Xist* mutation, suggesting that the primary imprint for imprinted XCI, which is likely to be H3K27me3 modification in maternal *Xist* locus^12^, might be erased in ExE cells^46^. Oikawa *et al*. also demonstrated that the primary imprint mark must be erased in the TE/ExE cells of E4.5 embryos by nuclear transfer experiments^10^. These results suggest that imprinted XCI is somehow maintained during ExE development, although its regulatory mechanism might be converted to a different one. The timing of the transient loss of *Xist* in ExE cells, shown in this study, roughly matches the transition period of the mechanism for imprinted XCI regulation in ExE cells suggested by Oikawa *et al*.^10^. Therefore, we speculate that the transient loss of *Xist* observed in the ExE might be involved in the conversion of the imprinted XCI regulatory mechanism in the ExE lineage.

## Methods

### Mice

The mouse strain C57BL/6J was used throughout the experiments. As an exception, embryos with mixed genetic background, BDF1xC57BL/6J, were used for *Lamp2* and *Pgk1* RNA-FISH experiments, because only very faint or no FISH signals were detected around E4.75 when we used embryos of C57BL/6J strain. E3.5–5.5 embryos were recovered at intervals of 6 h from naturally mated female mice. Noon was set as E0.5 on days when vaginal plugs were detected. We collected embryos between E3.5 and E4.0 by flushing the uteri. In many studies, E4.5 stage embryos were obtained by culturing preimplantation blastocyst or by flushing out the uteri. However, we experienced that the number of embryos collected by flushing out around E4.5 stage is substantially smaller than those obtained at E3.5, indicating that embryos firmly attached (=implanted) to the uterus cannot be recovered by flushing out, and it is likely that only embryos of relatively early stage (not yet firmly attached) were collected. Therefore, embryos after E4.25 were collected by manual dissection. The collected embryos were used immediately for the subsequent analysis without embryo culture. All animal experiments were performed in accordance with the guidelines for the experimental use and care of laboratory animals of the RIKEN Tsukuba Institute with prior approval from the Institutional Animal Experiment Committee of the RIKEN Tsukuba Institute.

### Strand-specific probe synthesis for whole-mount 3D RNA-FISH

Strand-specific DNA probes for detecting the *Xist*, *Tsix*, *Lamp2 and Pgk1* RNAs were prepared according to our published protocol^47^. For details, see the Supplementary Methods.

### Expression analysis of *Xist*/*Tsix* via whole-mount 3D RNA-FISH combined with immunofluorescence

Whole embryos were dissected then treated with 0.1% Triton X-100 in phosphate-buffered saline (PBS) for 10 s on ice for permeabilization. After fixation with 4% paraformaldehyde in PBS with 0.1% Triton X-100 for 10 min at room temperature, RNA-FISH and immunofluorescence analyses were performed. Details of the procedure, including the antibodies used, are provided in the Supplementary Methods.

### Analysis of *Xist*/*Tsix* expression patterns

*Xist* and *Tsix* expression patterns in individual cells from each lineage were examined as follows. The whole embryo was subjected to *Xist*/*Tsix* RNA-FISH followed by immunostaining with a marker that was specific to a particular lineage: POU5F1 or NANOG for ICM/embryonic ectoderm, GATA6 for PE/VE, and CDX2 for TE/ExE. Images were acquired using confocal microscopy. Optical sections of the confocal images were examined manually, and *Xist*/*Tsix* expression patterns in each cell of the embryos were determined by considering the overlaps of cellular images between the z-stack images. *Xist* expression patterns in each cell were classified into four categories: (1) cells with one *Xist* cloud per nucleus; (2) cells with single pinpoint or dispersed signals; (3) cells with two *Xist* signals; or (4) cells with no *Xist* signal. *Tsix*-positive cells were categorized into cells with two, one, or no signals. The combinations of *Xist* and *Tsix* expression patterns in each cell were determined, and the proportion of each combination was calculated and shown as a matrix. We determined *Xist* and *Tsix* expression patterns and their combinations in almost all the cells that constituted a whole embryo at a particular stage. We performed these analyses for nine different embryonic stages and for three different cell lineages of each stage. We repeated these experiments at least three times for each embryonic stage. At E6.5, more than 250 embryonic ectoderm cells in total were randomly chosen from two embryos (115 and 139 cells, respectively) and their *Xist* and *Tsix* expression patterns were examined. Confocal image data used in this study have been deposited to Systems Science of Biological Dynamics (SSBD) database (http://ssbd.qbic.riken.jp) and will be open to the public.

### Immunofluorescence analysis of epigenetic modifications

Whole embryos were dissected, treated with 0.1% Triton X-100 in PBS for 10 s on ice for permeabilization, and fixed with 4% paraformaldehyde in PBS with 0.1% Triton X-100 for 10 min at room temperature followed by immunofluorescence analysis. For details, including antibody information, see the Supplementary Methods.

## Supplementary information

Supplementary Information

Supplementary Video S1

Supplementary Video S2
