# Supplementary material for: Xist/Tsix expression dynamics during mouse peri-implantation development revealed by whole-mount 3D RNA-FISH
Source: Sci Rep. 2019 Mar 6;9:3637. doi: 10.1038/s41598-019-38807-0 (PMC6403393; doi:10.1038/s41598-019-38807-0)
Supplement: Supplementary file 1 — Supplementary Information [file 41598_2019_38807_MOESM1_ESM.pdf]

Supplementary Information

Xist/*Tsix* expression dynamics during mouse peri-implantation development revealed by whole-mount 3D RNA-FISH

Hirosuke Shiura [1,2,3] and Kuniya Abe [1,4] *

1 Technology & Development Team for Mammalian Genome Dynamics, RIKEN BioResource Research Center, 3-1-1 Koyadai, Tsukuba, Ibaraki 305-0074, Japan
2 Department of Epigenetics, Medical Research Institute, Tokyo Medical and Dental University (TMDU), 1-5-45 Yushima, Bunkyo-ku, Tokyo 113-8510, Japan
3 Faculty of Life and Environmental Sciences, University of Yamanashi, 4-4-37 Takeda, Kofu, Yamanashi 400-8510, Japan
4 Graduate School of Life and Environmental Sciences, University of Tsukuba, Ibaraki 305-8577, Japan

*Corresponding author: Kuniya Abe, Technology & Development Team for Mammalian Genome Dynamics, RIKEN BioResource Research Center, 3-1-1 Koyadai, Tsukuba, Ibaraki 305-0074, Japan
Tel: +81-29-836-9198; Fax: +81-29-836-9199; E-mail: kuniya.abe@riken.jp

Supplementary Methods

Strand-specific probe synthesis for whole-mount 3D RNA-FISH

Strand-specific DNA probes for detecting the *Xist, Tsix, Lamp2 and Pgk1* RNAs were prepared according to our published protocol [1]. Single-stranded RNAs of the same strand as *Xist, Tsix, Lamp2 and Pgk1* corresponding to fragments of the *Xist* RNA (exon 1; 1986–5786 bp; NR_001463), *Tsix* RNA (intron 3; 5787–9498 bp; NR_001463), *Lamp2* (ChrX: 38,448,352–38,452,813 (GRCm38/mm10)) and *Pgk1* (ChrX: 106,187,703-106,191,342 (GRCm38/mm10)) were synthesized by *in vitro* transcription using T7 or T3 RNA polymerases (Roche, Mannheim, Germany). Using the *in vitro*-transcribed RNA as a template, a fluorescence-labelled DNA probe was generated by random-primed reverse transcription (RT) using SuperScript III reverse transcriptase (Life Technologies Corporation, Carlsbad, CA, USA). Before the RT reaction, 7.5 μL of the RNA/primer mix containing 4 μg of synthesized RNA and 15 μg of random hexamers (Life Technologies Corporation) was denatured by incubation at 65 °C for 5 min and immediately placed on ice for at least 5 min. The RT reaction mix was composed of 4 μL of 5× First-Strand Buffer, 2 μL of 0.1 M DTT, 1 μL of RNase OUT (40 U/μL), 1 μL of SuperScript III RT (200 U/μL), and 4.5 μL of fluorescence-labelled dNTP mixture. The fluorescence-labelled dNTP mixture used for *Tsix*, *Lamp2* and *Pgk1* probes was prepared by combining 3.5 μL of 1 mM Cy3-dCTP (GE Healthcare, Chicago, IL, USA; PA53021) and 1 μL of a dNTP mixture containing dATP, dTTP, and dGTP (5 mM each) and dCTP (1.5 mM). For the *Xist* probe, 1.7 μL of 1 mM Green-dUTP (Abbott Molecular, Des Plaines, IL, USA, 02N32-050), 1.8 μL of RNase-free water, and 1 μL of a dNTP mixture containing dATP, dCTP, and dGTP (5 mM each) and dTTP (3.3 mM) were combined and used. The RNA/primer mix described above was mixed with 12.5 μL of the RT reaction mix and the reaction was incubated at 25 °C for 10 min, 50 °C for 90 min, and 70 °C for 15 min. After the RT reaction, the RNA that remained in the reaction was destroyed by adding 2 μL of 4 M NaOH followed by incubation at 37 °C for 30 min. The probe solution was neutralized by adding 2 μL of 4 M HCl. After ethanol precipitation using ammonium acetate, the probe DNA was dissolved in 20 μL of formamide and stored at 4 °C in the dark.

Expression analysis of *Xist*/*Tsix* via whole-mount 3D RNA-FISH combined with immunofluorescence

Whole embryos were dissected, treated with 0.1% Triton X-100 in PBS for 10 s on ice for permeabilization, and fixed with 4% paraformaldehyde in PBS with 0.1% Triton X-100 for 10 min at room temperature. After washing with PBS containing 0.1% Triton X-100 for 5 min at room temperature, the samples were incubated sequentially in 2 × SSC buffer with 0.05% Tween 20 for 10 min; 2 × SSC and 25% formamide with 0.05% Tween 20 for 10 min; and 2 × SSC and 50% formamide with 0.05% Tween 20 for 10 min twice. The probe mix was prepared by mixing 15 μL of hybridization solution (4 × SSC, 4 mg/mL of bovine serum albumin (BSA)), 10 μL of formamide, 3 μL of 1 μg/μL of mouse Cot-1 DNA (Life Technologies Corporation) (dissolved in formamide), 1 μL of the *Xist* probe, and 1 μL of the *Tsix* probe. Before the hybridization step, the probe mix was incubated at 70 °C for 10 min and then placed on ice for 5 min (for denaturation). After prehybridization in hybridization buffer (2 × SSC, 2 mg/mL of BSA, and 50% formamide) for 20 min, the embryo samples were placed into a 30 μL drop of the probe mix on a 35 mm Petri dish and then incubated for 8–15 h at 37 °C for hybridization. The drop containing the samples was covered with liquid paraffin to prevent evaporation. After hybridization, the samples were washed twice with a solution containing 2 × SSC, 50% formamide, and 0.05% Tween 20 for 5 min at 37 °C; twice with 2 × SSC containing 0.05% Tween 20 for 5 min at 37 °C; and then with PBS containing 0.05% Tween 20 (PBST) for 5 min at room temperature. The samples were refixed with 4% paraformaldehyde in PBST for 5 min, washed with PBST for 5 min at room temperature, and then subjected to immunofluorescence experiments. After incubation in blocking buffer (3% BSA and 5% donkey serum in PBST) for 30 min, the samples were reacted with the primary antibody in blocking buffer at room temperature for 90–180 min or at 4 °C overnight: for POU5F1, 1:300 goat polyclonal anti-Oct-3/4 (N-19; Santa Cruz Biotechnology Inc., Dallas, TX, USA; sc-8628); for NANOG, 1:100 rabbit polyclonal anti-Nanog (ReproCELL; Boston, MA, USA; RCAB0001P); for GATA6, 1:200 goat polyclonal anti-Gata6 (R&D Systems Inc., Minneapolis, MN, USA; BAF1700); and for CDX2, 1:200 rabbit polyclonal anti-Cdx2 (Abcam, Cambridge, MA, USA; ab88129). After washing with PBST for 5 min three times, the samples were incubated with the secondary antibody conjugated with Alexa Fluor 647 in the blocking buffer at room temperature for 90–180 min or at 4 °C overnight: for POU5F1 and GATA6, 1:500 Alexa Fluor 647 donkey anti-goat IgG (H+L) (Life Technologies

Corporation; A-21447); for NANOG and CDX2, 1:500 Alexa Fluor 647 donkey anti-rabbit IgG (H+L) (Life Technologies Corporation; A-31573). After PBST rinsing and post-fixation in 4% paraformaldehyde in PBST, the nuclear DNA of the samples was stained with DAPI and the samples were imaged using a confocal fluorescence microscope (LSM780; Carl Zeiss, Jena, Germany). Optical sections of whole embryos were imaged at intervals of 0.8 μm and were used to reconstruct images.

Immunofluorescence analysis of epigenetic modifications

Whole embryos were dissected, treated with 0.1% Triton X-100 in PBS for 10 s on ice for permeabilization, and fixed with 4% paraformaldehyde in PBS with 0.1% Triton X-100 for 10 min at room temperature. After incubation with PBS containing 0.1% Triton X-100 for 60 min at 4 °C, samples were pre-blocked in blocking buffer (3% BSA and 5% donkey serum in PBST) for 30 min, and then reacted with the primary antibody in blocking buffer for 3–4 days at 4 °C: for 5mC, 1:5000 mouse monoclonal anti-5mC (Eurogentec; BI-MECY-0100); for 5hmC, 1:2000 rabbit polyclonal anti-5hmC (ACTIVE MOTIF; 39769); for H3K9me2, 1:500 rabbit polyclonal anti-H3K9me2 (Millipore; 07-441); for H3K9me3, 1:500 rabbit polyclonal anti-H3K9me3 (Millipore; 07-523) and for H3K27me3, 1:2000 rabbit polyclonal anti-H3K27me3 (Millipore; 07-449). Before reaction with primary antibody against 5mC and 5hmC, the samples were treated with 4N HCl with 0.05% Tween 20 for denaturing of genomic DNA for 10 min at room temperature, and neutralized in 1M Tris-HCl (pH8.0) with 0.05% Tween 20 for 10 min at room temperature. After reaction with primary antibody, the samples were washed with PBST for 5 min three times and incubated with the secondary antibody conjugated with Alexa Fluor 488 or 555 in the blocking buffer for 3–4 days at 4 °C: for 5mC, 1:500 Alexa Fluor 488 donkey anti-mouse IgG (H+L) (Life Technologies Corporation; A-21202); for 5hmC, H3K9me2, H3K9me3 and H3K27me3, 1:500 Alexa Fluor 555 donkey anti-rabbit IgG (H+L) (Life Technologies Corporation; A-31572). After PBST rinsing and post-fixation in 4% paraformaldehyde in PBST, the nuclear DNA of the samples was stained with DAPI and the samples were imaged using a confocal fluorescence microscope (LSM780; Carl Zeiss, Jena, Germany).

Reference

1 Shiura, H., Sakata, Y., Abe, K. & Sado, T. RNA-FISH and Immunofluorescence of

Mouse Preimplantation and Postimplantation Embryos. *Methods in molecular biology* **1861**, 161-176 (2018).

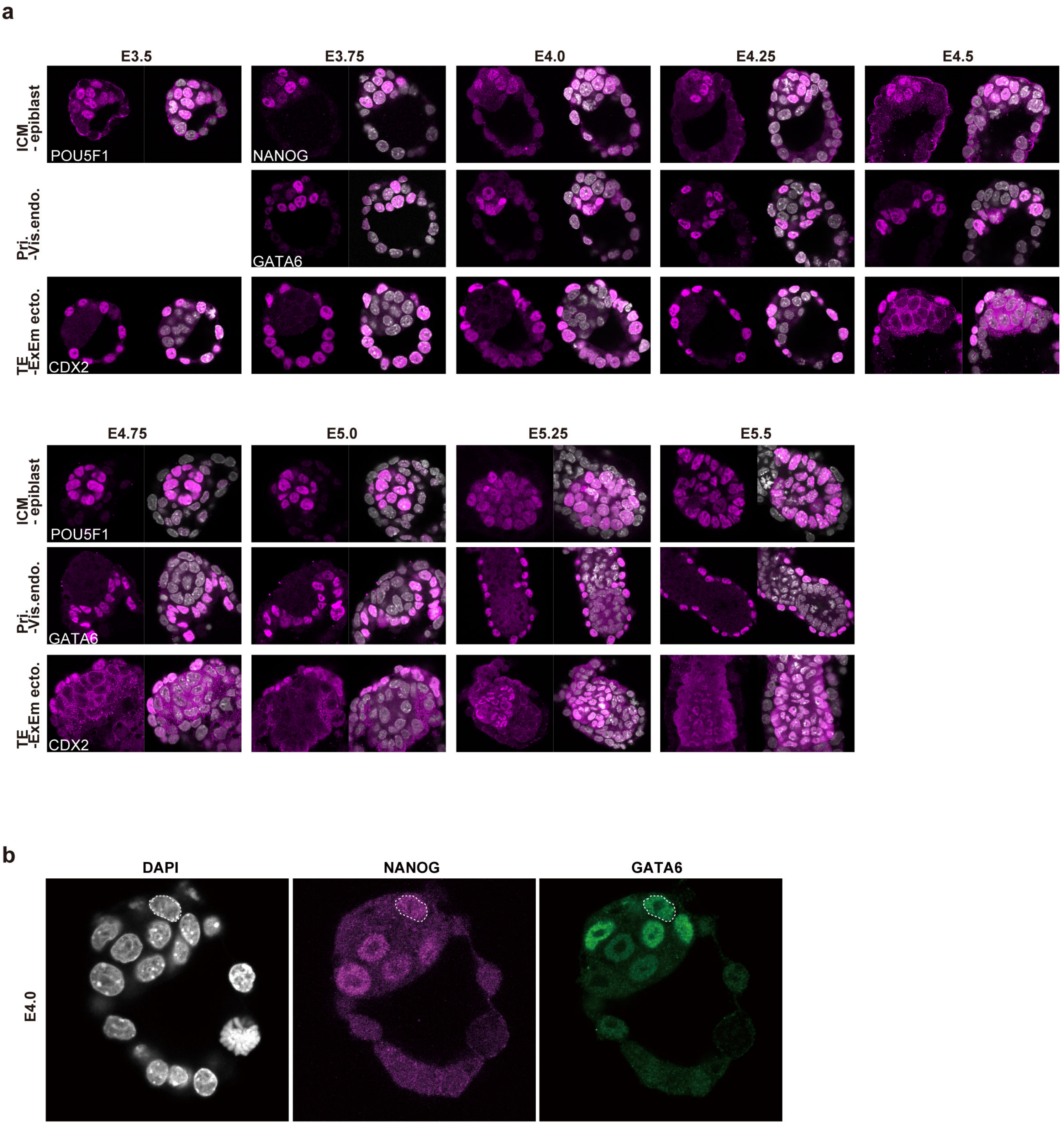

Supplementary Figure S1: Immunofluorescence images of the markers of each lineage expressed during peri-implantation development.

(a) Representative images of lineage-marker expression. Whole embryos were stained with lineage marker antibodies (magenta) against ICM/epiblast (POU5F1 or NANOG (upper panel)), primitive/visceral endoderm (GATA6 (middle panel)), and TE/extraembryonic ectoderm (CDX2 (lower panel)). Images of the marker alone (left) and of the marker with nuclear staining (white) are shown for each stage and marker combination.

(b) NANOG and GATA6 expression in an E4.0 embryo. The dotted circle indicates a cell showing both NANOG and GATA6 expression.

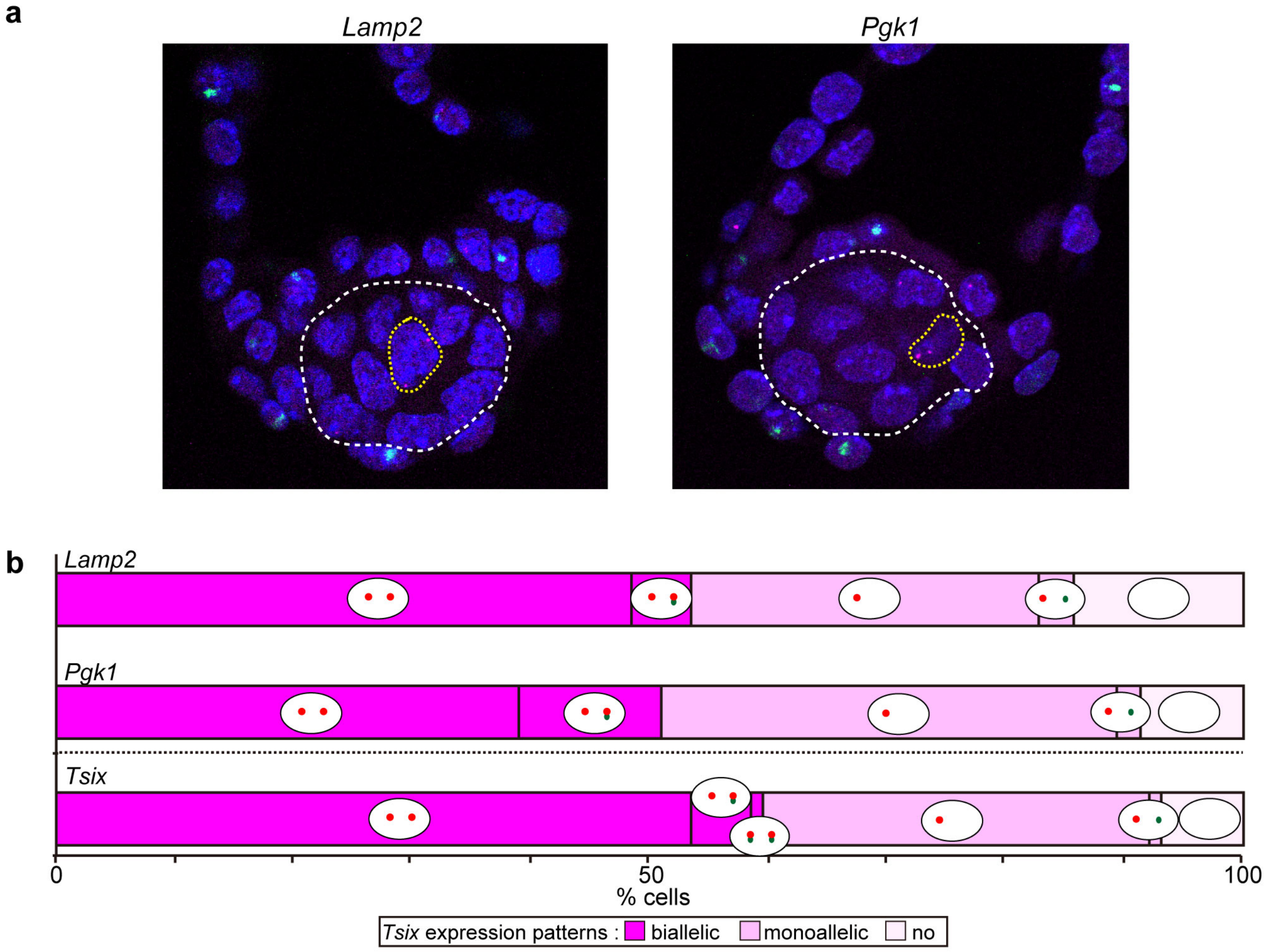

Supplementary Figure S2: Additional information for RNA-FISH analysis of *Lamp2* and *Pgk1* at E4.75.

(a) Representative images of *Xist/Lamp2* (left) and *Xist/Pgk1* (right) RNA-FISH at E4.75. The green, magenta and blue colors indicate *Xist*, *Lamp2* or *Pgk1* expression and nuclear DNA staining, respectively. In each image, ICM/epiblast is enclosed by a dotted white circle and the cell showing biallelic expression of *Lamp2* or *Pgk1* is enclosed by yellow circle.

(b) The expression patterns of *Lamp2, Pgk1* or *Tsix* combined with *Xist* in the embryonic cell lineage at E4.75. In this stage, re-expression of *Xist* was observed in a small subset of cells.

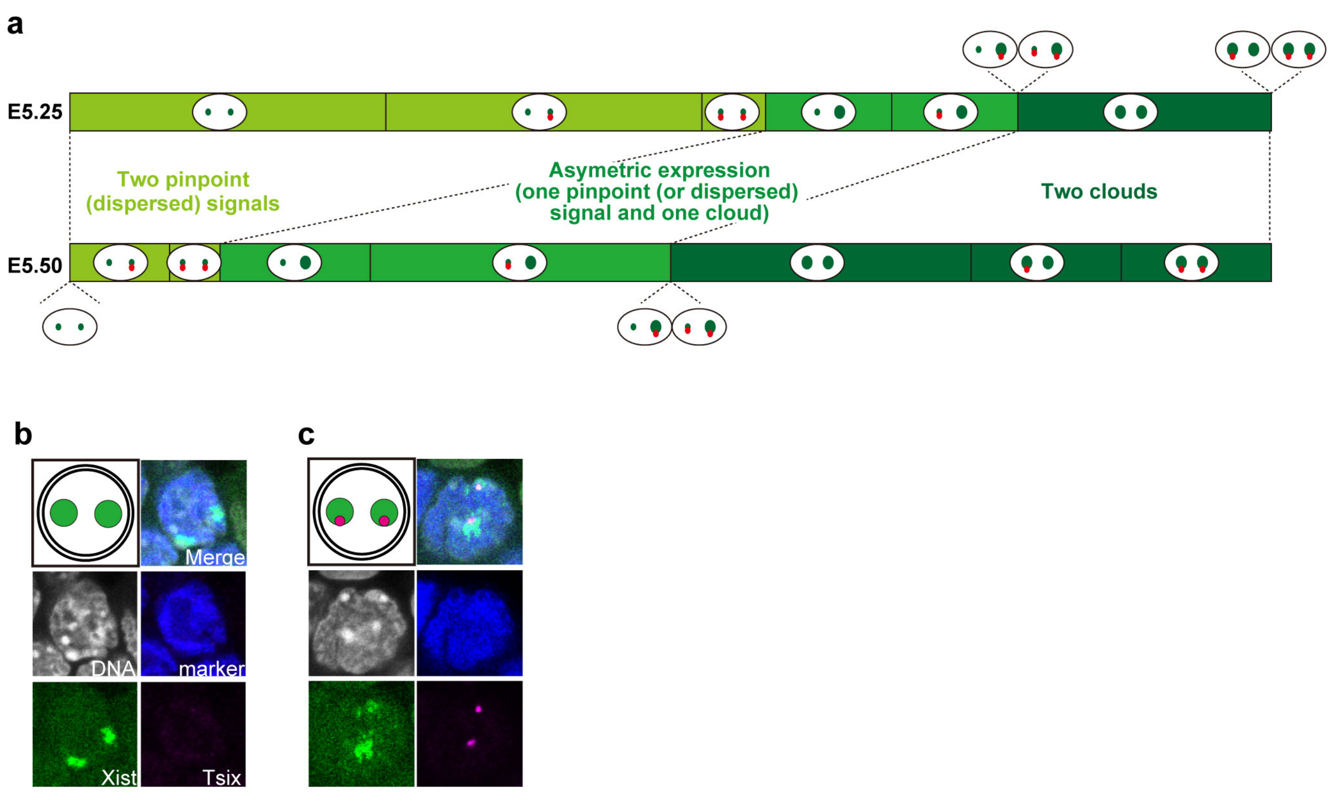

Supplementary Figure S3: Detailed distribution of cells showing two *Xist* signals.

(a) Cells showing two *Xist* signals in embryonic lineage at E5.25 and 5.50 are categolized based on *Xist* expression patterns (two pinpoints (or dispersed) signals, one pinpoint (or dispersed) signal and one cloud, and two clouds, and the proportions of each category are shown.

(b)(c) Representative images of cells showing two *Xist* cloud (green) other than Fig. 1g. The blue, magenta and white colors indicate lineage-marker (POU5F1) expression, Tsix and nuclear DNA staining, respectively. (b) Two *Xist* clouds and no *Tsix* signal, and (c) two *Xist* clouds and two *Tsix* signals.

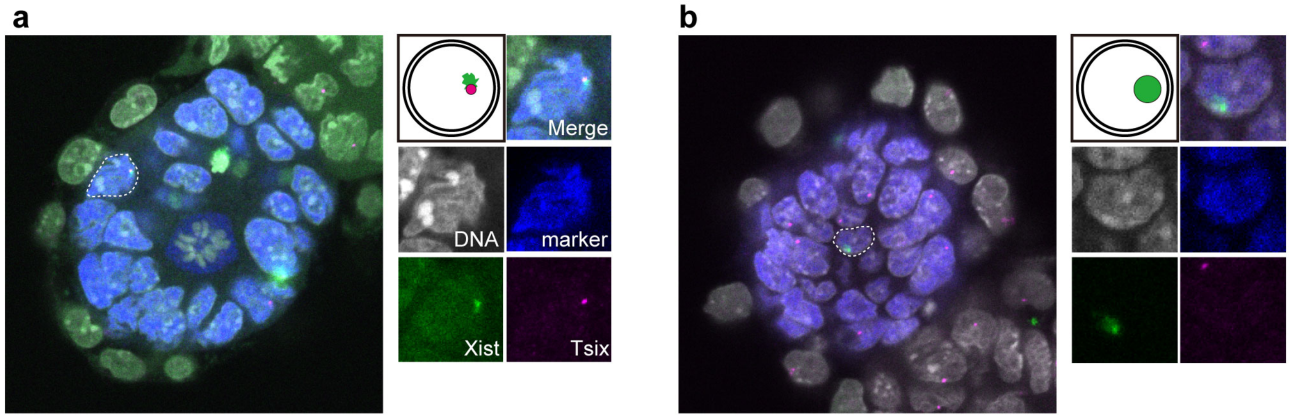

Supplementary Figure S4: Representative images of *Xist* positive male cells.
(a)(b) Male cells exhibiting *Xist* signal in embryonic lineage at E5.5. The blue, magenta and white colors indicate lineage-marker (POU5F1) expression, *Tsix* and nuclear DNA staining, respectively. (a) One *Xist* dispersed signal and *Tsix* signal, and (b) one *Xist* cloud. The cells enclosed by a dotted circle correspond to the cells shown in small panels.

Supplementary Video caption

Supplementary Video S1 and S2

Examples of the whole-mount RNA-FISH images acquired from E5.5 and E3.5 embryos.

The whole-mount RNA-FISH images were obtained from E5.5 (S1) and E3.5 (S2) embryos. The green signals represent *Xist*, whereas the red signals correspond to the *Tsix* RNA. Immunofluorescence against POU5F1 (S1) or CDX2 (S2) (blue) and nuclear staining (white) are also shown.
